# Hearing Loss due to Carbon Monoxide Poisoning

**DOI:** 10.1155/2013/940187

**Published:** 2013-05-15

**Authors:** Amir Houshang Mehrparvar, Mohammad Hossein Davari, Abolfazl Mollasadeghi, Mohammad Reza Vahidi, Mehrdad Mostaghaci, Maryam Bahaloo, Pedram Shokouh

**Affiliations:** ^1^Department of Occupational Medicine, Shahid Sadoughi University of Medical Sciences, Farrokhi Street, Yazd 89138-14389, Iran; ^2^Department of Otorhinolaryngology, Shahid Sadoughi University of Medical Sciences, Yazd 89138-14389, Iran; ^3^Cardiovascular Research Center, Isfahan Cardiovascular Research Institute, Isfahan University of Medical Sciences, Isfahan 81465-1148, Iran

## Abstract

Carbon monoxide poisoning is one of the rare causes of hearing loss which may cause reversible or irreversible, unilateral or bilateral hearing loss after acute or chronic exposure. In this report, we present a case of bilateral sensorineural hearing loss in a secondary smelting workshop worker after an acute exposure to carbon monoxide. This complication was diagnosed by pure-tone audiometry and confirmed by transient evoked otoacoustic emissions. Hearing loss has not improved after 3 months of followup.

## 1. Introduction

Carbon monoxide (CO) is a colorless, odorless, and nonirritant gas which is a common cause of mortality due to acute poisoning [1]. This gas is a by-product of incomplete combustion of hydrocarbons. The most common source of environmental exposure to CO is smoking followed by inadequate ventilation [2]. Affinity of CO for hemoglobin is 200 times that of oxygen. Bonding of CO with hemoglobin results in the production of carboxyhemoglobin, which reduces blood oxygen-carrying capacity, competes with oxygen at the heme binding sites, and shifts the oxygen hemoglobin dissociation curve to the left [3]. CO exposure also may cause inflammation through mechanisms independent of hypoxia, resulting in neurologic and cardiac injuries [4].

The symptoms of CO poisoning are nonspecific [5]. Acute mild exposure to CO leads to headache, myalgia, dizziness, and neurologic disturbance [1, 6], while heavier exposure may lead to retinal hemorrhage, myocardial infarction [2], loss of consciousness, coma, and death [4]. After CO poisoning, patients may suffer from some neurologic sequelae such as motor disturbances, peripheral neuropathy, and hearing loss [4]. In contrast, chronic exposure may produce different symptoms comprising fatigue, memory loss, sleep disturbance, vertigo, and hearing loss [7, 8]. Sensorineural hearing loss is among the complications that may be caused by either acute or chronic exposure to CO.

## 2. Case Presentation

Our case was a 22-year-old male working in a secondary smelting workshop in a rotating shift schedule. He was healthy without any history of hearing loss in his preplacement evaluations. 

The night before his admission, he had worked the night shift of the factory. In the morning, his coworkers found him unconscious. Immediately, he was brought unconscious to the hospital. After intubation, fluid therapy was begun, and he was transferred to the intensive care unit. His vital signs at admission were as follows: pulse rate: 115 beats/min; respiratory rate: 14/min; blood pressure: 115/80 mmHg; temperature: 37.3°C. His Glasgow Coma Scale at that time was 10. The results of arterial blood gas analysis taken at admission are presented in Table 1. 

After history taking, the diagnosis of CO poisoning was suggested for him, and oxygen therapy via face mask (15 lit/min) was immediately started. Hyperbaric O_2_ therapy was not available in that hospital center at the time. 

Chest X-ray showed unilateral consolidation and signs of pulmonary edema. His brain computed tomography scan demonstrated mild brain edema.

**Figure 1:**
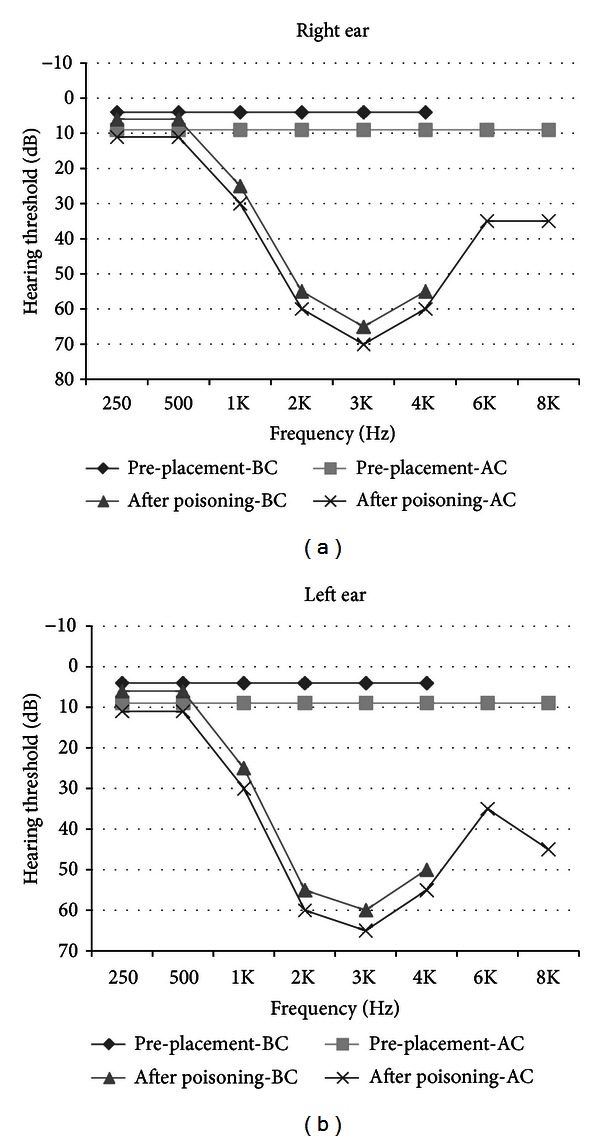
Patient's puretone audiograms in pre-placement evaluation and after CO poisoning (BC: bone conduction; AC: air conduction).

The patient became conscious after 4 days. After consciousness, he complained of hearing impairment without vertigo or tinnitus. He did not have any history of hearing loss, head trauma, loud noise exposure, and exposure to ototoxic drugs or substances. External ear canal and tympanic membrane examination was normal. Afterwards, the assessment of his auditory system was performed using pure-tone audiometry (PTA), tympanometry, and transient evoked otoacoustic emissions (TEOAEs). The PTA showed bilateral mid- to high-frequency sensorineural hearing loss.  Figure 1 shows his audiograms in preplacement evaluations and after the poisoning. Speech reception threshold was in accordance with the PTA findings. TEOAEs were absent in both ears. 

He was discharged from the hospital after 8 days without any complications except for the hearing loss. Before being discharged, a written informed consent was obtained from the patient for the presentation of his disorder. Audiometric assessments after 1, 2, and 3 months did not show any improvements in his hearing level. 

## 3. Discussion

Acute or chronic exposure to CO is one of the known but rare causes of hearing loss [8]. This kind of impairment is caused by the effect of CO on blood oxygen-carrying capacity [9] or free radical production [10]. It has been evidenced that auditory cortex, inferior colliculus, and cochlea are very sensitive to anoxia, but central auditory pathways are more sensitive than the cochlea [11]. Hearing impairment due to anoxia could be either reversible or irreversible [12]. This type of hearing loss is usually bilateral and variable in severity [13]. 

CO-induced hearing loss mostly affects high frequencies, but further exposure to CO may affect low frequencies as well. If it is reversible, improvement is observed first in low frequencies [9]. Consistent with the findings of Pillion [13] and Skrzypczak et al. [14], we observed a bilateral, high-frequency sensorineural hearing loss, and considering TEOAEs, the location of injury was in the outer hair cells. Nevertheless, in a case reported by Lee et al., hearing loss involved all frequencies except for 4 KHz [8]. It should be mentioned that unilateral hearing loss due to CO poisoning has also been observed and reported [15, 16]. Of note, a mid-frequency notch in PTA has been discerned by some authors [17, 18], but not by others [13, 15]. 

After 3 months of followup, there were not any detectible improvements in the patient's hearing condition. Unlike our patient and the cases reported by Pillion [13] and Skrzypczak et al. [14], hearing loss after CO poisoning has been reported to revert by Lee et al. [8], Shahbaz Hassan et al. [18], Razzaq et al. [15], and Michalska-Piechowiak et al. [16]. In line with other similar cases [8, 11], our patient has never complained of tinnitus or vertigo.

Hearing evaluation is not routinely performed in patients who suffer from CO poisoning. This case showed that hearing assessment should be integrated into the clinical evaluations of brain injury due to CO poisoning. 

## Figures and Tables

**Table 1:** Results of the arterial blood gas analysis of the patient at admission and after 24 hours.

Parameters	At admission	After 24 hours
Hemoglobin (g/dL)	13	13.6
Temperature (°C)	37	37
pH	7.40	7.47
PCO_2_ (mmHg)	27	32
PO_2_ (mmHg)	37	110
BE (mEq/L)	−7	−0.1
BEecf (mEq/L)	−8.5	−0.9
BB (mEq/L)	40	47.3
HCO_3_ (mEq/L)	16	22.8
O_2_ saturation (%)	69	98.6

BEecf: base excess in the extracellular fluid; BB: buffer base.
